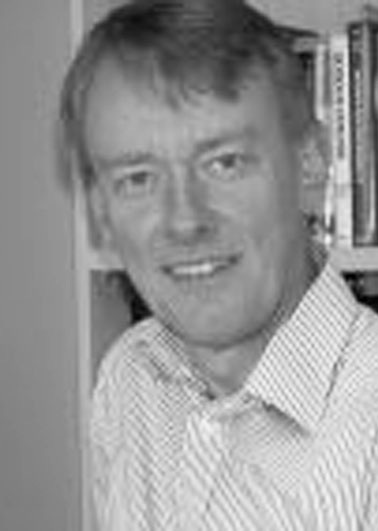# Trends and Prospects of Bimetallic Exsolution

**DOI:** 10.1002/chem.202004950

**Published:** 2021-02-24

**Authors:** Chenyang Tang, Kalliopi Kousi, Dragos Neagu, Ian S. Metcalfe

**Affiliations:** ^1^ School of Engineering. Newcastle University Newcastle upon Tyne NE1 7RU UK; ^2^ Department of Process and Chemical Engineering University of Strathclyde Glasgow G1 1XL UK

**Keywords:** bimetallic exsolution, catalysis, electrochemistry, energy conversion, nanomaterials

## Abstract

Supported bimetallic nanoparticles used for various chemical transformations appear to be more appealing than their monometallic counterparts, because of their unique properties mainly originating from the synergistic effects between the two different metals. Exsolution, a relatively new preparation method for supported nanoparticles, has earned increasing attention for bimetallic systems in the past decade, not only due to the high stability of the resulting nanoparticles but also for the potential to control key particle properties (size, composition, structure, morphology, etc.). In this review, we summarize the trends and advances on exsolution of bimetallic systems and provide prospects for future studies in this field.

## Graphical abstract

**Exsolved bimetallic nanoparticles** have attracted a lot of attention because they combine high stability and potential to control key particle properties (size, composition, structure, morphology, etc.) endowed by the exsolution method with additional unique properties mainly originating from the synergistic effects between the two different metals. These materials have enabled step‐change advances across a wide range of applications including electrochemical energy conversion and heterogenous catalysis. In this review the trends and advances of exsolution of bimetallic systems are presented and future directions of research provided.

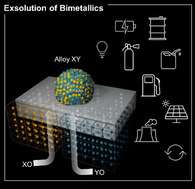

## 1 Introduction

Metal nanoparticles dispersed on solid oxide supports are increasingly applied in a wide range of applications such as heterogeneous catalysis, electrochemical conversion and photocatalysis. Bimetallic nanoparticles, composed of two metal species, often show distinct catalytic properties and stability as compared to individual metals, which is typically ascribed to changes in the electronic and/or geometric structures that occur when the metals are combined.^[1]^ Bimetallics are usually prepared by assembly or co‐deposition methods and varying their particle size, composition (ratio of metals involved), structure (alloys, core–shells, heterostructures, etc.) and morphology, can lead to a wide range of functional materials.^[2]^ In spite of this variety, bimetallics prepared through such methods often display limited long‐term stability, generally due to the weak metal‐support interactions. One way to stabilize them is through the exsolution method.

Redox exsolution, an alternative to assembly and deposition methods, has the potential to produce supported metal nanoparticles with combined high activity and stability. In this method, active species are substituted in a perovskite oxide matrix and subsequently emerge as metal nanoparticles at the surface, normally driven by thermal^[3]^ or electrical reduction.^[4]^ Exsolved nanoparticles are epitaxially grown from the parent oxide and partially socketed in it. This results in a strained particle‐oxide interface^[5]^ which endows the nanoparticles with enhanced stability against sintering and can also enhance resistance to poisoning by carbon deposition and sulfur, as well as high activity.

**Figure 1:**
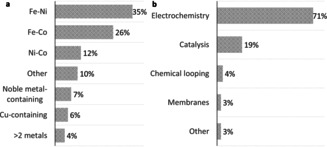
Statistic analysis on the papers published for bimetallic exsolution.

Co‐exsolution of two metals, while less studied than single metal exsolution, can lead to attractive structures and functionality, including synergistic effects between the two co‐exsolved metals.^[6]^ There are roughly 70 papers on bimetallic exsolution published in the past decade, from which statistical information on the compositions and applications of the exsolved bimetallics is extracted. The Fe‐containing compositions are the overwhelming majority followed by Ni‐based systems (Figure 1 a). However, other bimetallic compositions have also been occasionally reported, as well as alloys containing more than two metals and more complex heterostructures. Interestingly, there are no bimetallic materials containing two noble metals reported. The use of bimetallic exsolved systems spans over all kinds of applications including but not limited to electrochemistry (71 %), catalysis (19 %) and membranes (3 %) (Figure 1 b). Among these, electrochemistry[^6a^, ^6b^, ^6c^, ^7^] is the most common application but there are also a few studies focusing on catalytic applications such as methane conversion,^[8]^ CO oxidation^[9]^ or the water‐gas‐shift reaction.^[10]^ In spite of these exciting developments, to the best of our knowledge, there is no review that summarizes the advances on bimetallic exsolution. Here we aim to review the trends that underpin the exsolution of bimetallic systems in terms of design, tailoring and application and propose future directions for the development of the field.

## 2 Mechanism of Alloy Exsolution

**Figure 2:**
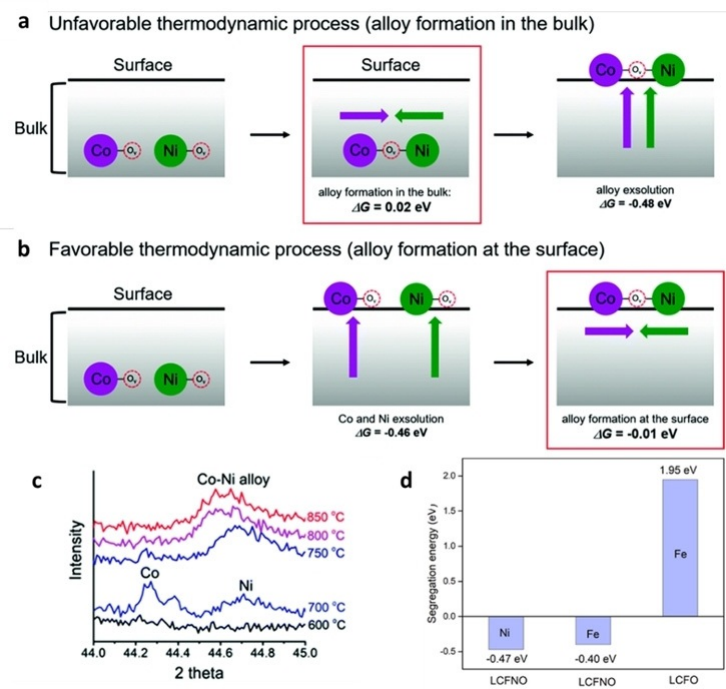
Schematic illustrations and energetics of alloy nanoparticle exsolution following: (a) bulk alloy formation, and (b) surface alloy formation mechanisms. (c) In situ X‐ray diffraction showing the changes of the exsolved phases on PrBaMn_1.7_Co_0.1_Ni_0.2_O_5+δ_ at different temperatures. Adapted with permission.^[11a]^ (d) Segregation energies of B‐site metals from LaCr_0.5_Fe_0.5_O_3_ (LCFO) and Ni‐doped LCFO (LCFNO). Adapted with permission.^[11d]^

Simultaneous exsolution of two or more reducible metal species from an oxide backbone will, in most instances, lead to the formation of alloy particles as evidenced by modelling studies.^[11]^ Generally, two possible exsolution mechanisms exist, namely the “bulk alloy formation” and “surface alloy formation” as schematically illustrated in Figure 2 a and b, respectively.^[11a]^ Exsolution is promoted by the oxygen vacancies introduced by reduction which are prone to move from the bulk towards the surface, hence it is favourable for exsolvable metals to segregate along with the oxygen vacancies (known as co‐segregation) due to the lower energy required.[^11a^, ^11b^] DFT calculations indicated that the co‐segregation of Ni, Co and Co−Ni with an oxygen vacancy are all thermodynamically favourable, due to their negative Gibbs energies of co‐segregation (−0.39, −0.53 and −0.48 eV, respectively). However, the Gibbs energies for metal aggregation in the bulk (0.02 eV) and at the surface (−0.01 eV) are both much more positive than those of metal co‐segregation. Thus, the energetics suggest that alloy formation in the bulk is less favourable than metal co‐segregation during the exsolution process and thus exsolution is more likely to follow the surface alloy formation mechanism due to the lower alloy formation energy at the surface.^[11a]^ This has also been demonstrated experimentally by employing in situ *x*‐ray diffraction where during reduction, and thus exsolution, separated Ni and Co peaks appeared first at a low temperature while the Co−Ni peak appeared at higher temperatures at the expense of the Ni and Co peaks (Figure 2 c).^[11a]^ Similar exsolution behaviour has also been observed for Co−Fe alloys using in situ scanning transmission electron microscopy, where the Co‐based nanoparticles and Co−Fe alloys appeared in sequence with the increasing temperature during reduction.^[11c]^

It is worth mentioning that some metals, such as Fe, would probably not exsolve if they are the sole species on the B‐site in the perovskite lattice due to their high segregation energy, but it could be possible to exsolve them together with a second, substituted, metal (like Ni and Co) with a lower segregation energy. This is because in mixed cation systems, the Gibbs energy of reduction is a function of the strength of the metal‐oxygen bonds of both substituted metals and hence the energy can be decreased by introducing more reducible ions (Figure 2 d).[^11c^, ^11d^, ^11e^] Besides, it is also reported that doping of Co increases the total energy of the perovskite system and the Co−Fe bond would form more easily than the Fe−Fe bond due to the lower formation energy, which also accounts for the promoting effects of Co on the Fe exsolution.^[11f]^

## 3 Bimetallic Alloy Nanoparticle Systems

Generally, in perovskite‐based systems the A‐site stoichiometry dictates which types of exsolution can occur: irreversible or reversible (also called intelligent concept). In the latter, the original matrix is stoichiometric and thus upon exsolution the thermodynamically stable perovskite segregates into a less thermodynamically stable mixture of A‐site oxide and B‐site exsolved components. Thus, these phases could recombined, giving the exsolved nanoparticles the ability to re‐dissolve depending on the gas environment, which in turn leads to materials with high durability due to the fact that they can regenerate. This applies both for single metals^[12]^ as well as for bimetallic systems.[^6d^, ^7g^, ^7h^, ^10^, ^11c^, ^11e^, ^13^] For example, Fe−Co nanoparticles from La_0.3_Sr_0.7_Cr_0.3_Fe_0.6_Co_0.1_O_3‐δ_ were found to completely redissolve into the perovskite when re‐oxidized at 800 °C while they remained on the surface as transition‐metal oxide at 700 °C.^[7g]^ On the other hand, during exsolution from A‐site deficient perovskites which are thermodynamically metastable, a stabler A‐site stoichiometric perovskite forms alongside the exsolved particles which makes re‐dissolution less likely and thus this exsolution irreversible. Moreover, nanoparticles exsolved from A‐site deficient perovskites are partially socketed in the surface and less likely to redissolve into the perovskite lattice under re‐oxidation leading to coke resistant, highly active materials mainly due to the alignment and socketing between the support and the exsolved particles.[^3^, ^5^] For example, Ni−Co nanoparticles exsolved from La_0.7_Ce_0.1_Co_0.3_Ni_0.1_Ti_0.6_O_3_ did not redissolve when treated under oxidizing conditions, but demonstrated coking resistance and matched noble metal commercial catalysts in automotive exhaust reaction conditions.^[9c]^ The reversible/intelligent exsolution method accounts for about 15 % of the total papers published until today on bimetallic exsolution. Nevertheless both types are reviewed in detail in the following section depending on the chemistry of the exsolved particles.

### 3.1 Fe−Ni

**Figure 3:**
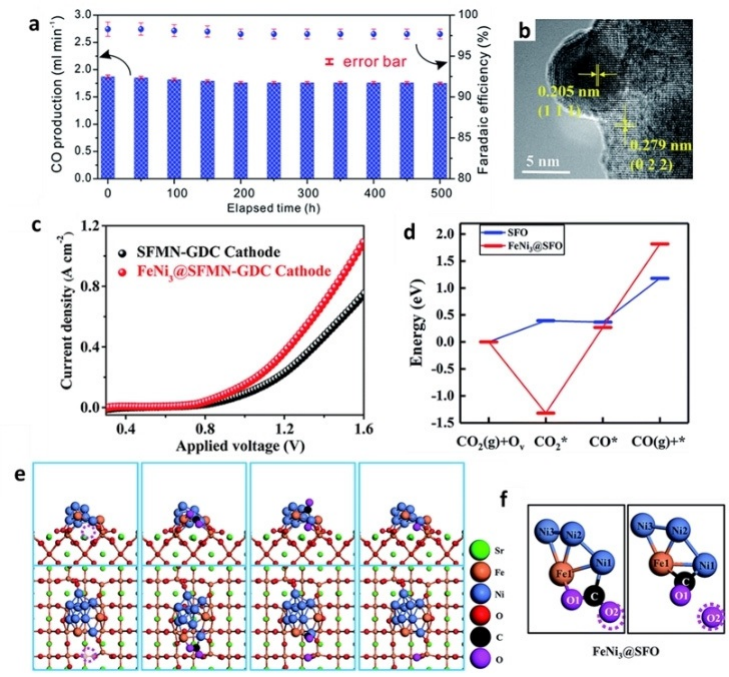
Exsolved Fe−Ni cathodes in CO_2_ electrolysis. (a) Cell performance over long‐term operation at 1.3 V and 800 °C. Adapted with permission.^[7b]^ (b) HRTEM image showing socketed interface between a exsolved Fe−Ni particle and a Sr_2_Fe_1.35_Mo_0.45_Ni_0.2_O_6‐δ_ substrate. (c) Enhanced CO_2_RR performances due to exsolution of Fe−Ni nanoparticles. DFT calculations showing (d) the energies of CO_2_RR over the perovskite surface and the interface between the exsolved Fe−Ni and the perovskite, and (e, f) the optimized reaction pathway at the metal‐support interface (magenta sphere and circle represent oxygen atom and vacancy involved in the reaction, respectively). Adapted with permission.^[14]^

Exsolved Fe−Ni alloys are frequently used as cathodes for CO_2_ or steam electrolysis because such cathodes demonstrate high catalytic activity, good stability under cell operation conditions and resistance to carbon deposition and nanoparticle sintering (Figure 3 a), due to the well‐known strong anchorage of the exsolved particles on the support (Figure 3 b).[^7a^, ^7b^, ^14^] For example, the interface between the exsolved Fe−Ni nanoparticles and the Sr_2_Fe_1.35_Mo_0.45_Ni_0.2_O_6‐δ_ substrate with abundant oxygen vacancies was found to promote the adsorption and activation of CO_2_, which resulted in much better performance for CO_2_ reduction reaction (CO_2_RR) as compared to the cathode based on the pristine perovskite (Figure 3 c–f).^[14]^ Similarly, such exsolved nanoparticle systems have been proven to have enhanced activity for water splitting since an electrolysis cell with a Sr_2_Fe_1.3_Ni_0.2_Mo_0.5_O_6_ cathode decorated with Fe−Ni exsolved nanoparticles demonstrated about twofold higher current density and half the electrode polarization resistance as compared to the cell using pristine cathode.^[15]^ A similar system (Sr_2_Fe_1.4_Ni_0.1_Mo_0.5_O_6‐δ_) was also reported to demonstrate excellent redox cycling stability via self‐regeneration of Fe−Ni nanoparticles, despite the fact that when re‐oxidized in air some Fe−Ni nanoparticles were oxidized to (Ni,Fe)O, remaining on the surface rather than reincorporate into the perovskite lattice.^[13a]^

Exsolved Fe−Ni alloys have demonstrated excellent catalytic activity, durability and resistance to coking and sulfur poisoning when used as anode materials for SOFCs.[^6a^, ^7c^, ^7d^] For instance, Sr_2_FeMo_0.65_Ni_0.35_O_6‐δ_ was found to partially decompose to a mixed Ruddlesden–Popper (RP) type layered Sr_3_FeMoO_7‐δ_, a perovskite Sr(FeMo)O_3‐δ_ and Fe−Ni nanoparticles, and the resulted anode showed high electronic conductivity, excellent catalytic activity and stability under both wet H_2_ and CH_4_.^[16]^

In addition, exsolved Fe−Ni nanoparticles were reported to improve hydrogen dissociative adsorption (rate‐limiting step) thus leading to increased catalytic activity.^[17]^ Interestingly, a general thermodynamic model was developed to predict the composition of those exsolved particles based on the approximation that the more reducible metal (Ni) completely exsolved while the content of the other metal (Fe) in the exsolved alloy is dependent on *P* ${}_{O{}_{2}}$ .^[17]^ Doping a small amount of alkali metals like Na on the A‐sites of a Sr_2_FeMo_0.65_Ni_0.35_O_6‐δ_ perovskite was found to facilitate the phase transition into a RP structure after reduction, increase the Ni content in the exsolved Fe−Ni alloys, and also introduce more surface oxygen vacancies ultimately leading to higher activity and enhanced coking resistance when employed in a SOFC anode under H_2_ and CH_4_.^[18]^ Lastly, exsolved Fe−Ni materials have also demonstrated potential to be used as electrodes for symmetrical SOFCs, as they exhibited improved activity for both fuel oxidation and oxygen reduction reaction, good durability and resistance to carbon deposition.^[19]^

**Figure 4:**
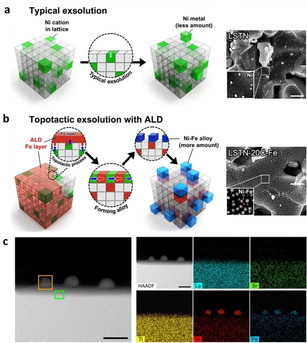
Formation of Fe−Ni alloyed nanoparticles via topotactic exsolution. (a, b) Schematic comparison between conventional and topotactic exsolution, and SEM images showing the exsolved particles for each case. (c) HAADF scanning TEM with EDS of the Fe−Ni alloyed nanoparticles via topotactic exsolution. Adapted with permission.^[20]^

Exsolved Fe−Ni alloy systems have also been employed as catalytic materials for reactions of methane reforming[^8a^, ^8b^] and CO oxidation.^[9a]^ For the former, the effect of the A‐site cations on the materials’ activity (LnFe_0.7_Ni_0.3_O_3‐δ_, Ln = La, Pr, Sm) was studied and it was found that PrFe_0.7_Ni_0.3_O_3‐δ_ composition resulted in the highest activity and stability due to the optimal composition of exsolved Fe−Ni nanoparticles (higher Fe content) and the redox properties of the oxide matrix.^[8a]^ In addition, an increase in the Ni doping level resulted in an increase in the oxygen deficiency of the perovskites due to the reduction of Fe^3+^/Fe^4+^ and Ni^2+^ species to lower cation valences which in turn enhanced Fe−Ni nanoparticle exsolution, resulting in improved activity for CH_4_ conversion (20 times over that of the pristine perovskite).^[8b]^ Fe−Ni exsolved catalysts have also been prepared through a so‐called “topotactic exsolution” method. Here, Fe was introduced as the guest cation (via infiltration or atomic layer deposition) on the surface of a Ni−containing perovskite and the exsolution process was promoted by ion exchange between Fe and Ni driven by the different segregation energies of the two metals.[^20^, ^21^] Thus, more Ni was dragged to the surface forming alloyed Fe−Ni nanoparticles with high population (Figure 4). This resulted in higher activity for methane dry reforming when compared to that of monometallic Ni catalyst prepared via normal exsolution while still maintaining excellent durability.[^20^, ^21^] For the latter, when used in CO oxidation, exsolved Fe−Ni nanoparticles from La_0.5_Sr_0.4_Fe_0.1_Ni_0.1_Ti_0.6_O_3_ exhibited high activity, good long‐term stability over 170 h and sulfur tolerance.^[9a]^

Finally, using Fe−Ni nanoparticles exsolved from Sr_0.9_(Fe_0.81_Ta_0.09_Ni_0.1_)O_3‐δ_ in a catalytic membrane reactor for methane partial oxidation, resulted in almost full CH_4_ conversion and nearly 100 % selectivity to CO and H_2_. Oxidising reaction conditions can cause exsolved nanoparticles to redissolve into the perovskite lattice (depending on the presence of A‐site deficiency) which can prove detrimental for their catalytic activity. Here, the oxygen‐permeable membrane could act as an oxygen distributor controlling oxygen partial pressure near the particles hence suppressing their dissolution in the perovskite during oxidation.^[8c]^

### 3.2 Fe−Co

Exsolution of Fe−Co nanoparticles has also been studied extensively usually as anode materials for SOFCs and similar to their Fe−Ni counterparts, they demonstrate a plethora of promising properties such as high activity, stability, coking resistance and sulfur tolerance.[^6b^, ^6c^, ^7e^, ^7f^, ^7g^] A Co‐doped La_0.6_Sr_0.4_FeO_3‐δ_ (LSF) anode was found to achieve high power density and lower polarization resistance in a SOFC cell as compared to its Mn‐doped counterpart due to the high catalytic activity of the exsolved Fe−Co particles towards hydrogen oxidation and the higher oxygen‐ion conductivity of the newly formed LaSrFeO_4_–La(Sr)Fe(Co)O_3_ (the perovskite phase of the Mn‐doped LSF was retained without formation of other phases after reduction).^[22]^ Similarly, an SOFC anode material comprising of Fe−Co nanoparticles distributed on RP‐type layered Sr_3_FeMoO_7_ was prepared by reducing Sr_2_FeMo_2/3_Co_1/3_O_6‐δ_ in hydrogen, resulting in greatly improved electrical conductivity and catalytic activity. Thus, the resulting SOFCs achieved high maximum power densities in H_2_ approximately 1.4 times higher than those of SOFCs employing a conventional Ni–Sm_0.2_Ce_0.8_O_1.9_ anode while at the same time also exhibiting high performance and coking resistance in C_3_H_8_.^[23]^ More interestingly, a double‐layered perovskite (Pr_0.4_Sr_0.6_)_3_(Fe_0.85_Mo_0.15_)_2_O_7_ decorated with Fe−Co nanoparticles, was used as the anode in a direct ethane‐fuelled proton‐conducting SOFC; high power density and high ethylene yield with ethylene selectivity of >91 % were achieved together with excellent stability and coking resistance in ethane.^[13b]^ A Pr_0.4_Sr_0.6_Co_0.2_Fe_0.7_Nb_0.1_O_3‐δ_ perovskite was employed as semiconducting material in a single‐layer fuel cell and, despite the lower fuel cell performance, it demonstrated enhanced stability as compared to the conventional lithiated metal oxides. Additionally, exsolution of Fe−Co alloy nanoparticles enhanced the performance of the starting perovskite, which improved electrode reaction kinetics, facilitated charge separation and ionic conduction.^[24]^

Notably, exsolution of metal particles is sometimes accompanied by a phase transition of the perovskite substrate. In this case, the exsolved nanoparticles usually contribute to high catalytic activity while the oxide substrate affects the pathway for electron/ion conduction, hence they are both important for determining the SOFC anode performance. However, a phase transition of the substrate is not always beneficial since studies have shown that extreme reducing conditions that promote the exsolution of Fe−Co particles and the phase transition of the perovskite substrates, can also result in decreased conductivity.^[25]^

The exsolved Fe−Co particle systems also find application in electrolysis and various symmetrical cells due to their activity for CO_2_RR^[11c]^ and oxygen evolution reaction (OER).^[26]^ For example, CO_2_ electrolysis performance of La_0.4_Sr_0.6_Co_0.2_Fe_0.7_Mo_0.1_O_3‐δ_ was enhanced after exsolution of Fe−Co nanoparticles, which seemed to originate from the metal–oxide interface that strengthened CO_2_ adsorption and activation. At the same time, the material also demonstrated good stability, coking resistance and redox recyclability.^[11c]^ Additionally, approximately 40 times higher activity for OER was demonstrated by an exsolved Fe−Co/LaCo_0.8_Fe_0.2_O_3‐δ_ system compared to the pristine perovskite. Operando X‐ray adsorption spectroscopy indicated that the enhancement in activity comes from the fact that exsolved nanoparticles self‐reconstructed into (Co/Fe)O(OH) with unsaturated coordination of metal ions in alkaline media during the OER, which acted as active species.^[26]^ Besides, exsolved Fe−Co materials have also been used as electrodes in symmetrical solid oxide cells both for the oxidation of different kinds of fuels (e.g., H_2_, LPG, C_2_H_5_OH and CH_3_OH) as well as for the electrolysis of H_2_O and/or CO_2_, exhibiting promising electrochemical performance and stability.[^13c^, ^27^]

**Figure 5:**
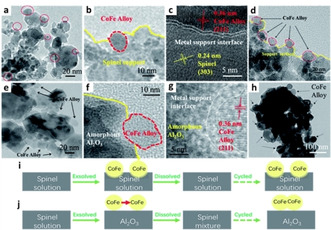
Redox stability of Fe−Co particles exsolved under different atmospheres. (a–c) Fe−Co particles freshly exsolved and (d) after 20 cycles when reduced in CO+CO_2_. (e–g) Fe−Co particles freshly exsolved and (h) after 20 cycles when reduced in CO. (i, j) Schematic illustration of the interface structure during redox cycles for exsolved particles induced by CO + CO_2_ and CO reduction, respectively. Adapted with permission.^[10]^

Last but not least, Fe−Co particles have also been used in chemical looping applications where CoFeAlO_*x*_ was designed as an oxygen carrier material for chemical looping water gas shift reaction, based on the known stability of alumina spinels and the high capacity and reactivity of cobalt ferrites. Here the redox cycle of the reaction caused exsolution and re‐dissolution of active Fe−Co particles. Interestingly, it was found that when reduced by mixed CO and CO_2_ the exsolved Fe−Co particles remained embedded in the support demonstrating high redox stability, while under CO, the spinel support was over‐reduced to Al_2_O_3_ and the metal‐support interface structure resembled a deposited‐like manner which led to easy sintering (Figure 5).^[10]^

### 3.3 Ni−Co

Ni−Co exsolution studies mostly refer to electrochemical applications,[^7h^, ^7i^, ^7j^] in which these particles demonstrate similar characteristics to those of their Fe based counterparts (Section 3.1 and 3.2). Recently, it was reported that an anode material based on Ni−Co alloy nanoparticles exsolved from La_0.52_Sr_0.28_Ti_0.94_Ni_0.03_Co_0.03_O_3‐δ_ exhibited high catalytic activity towards NH_3_ decomposition and H_2_ oxidation due to the abundant active sites and the balanced NH_3_ adsorption and N_2_ desorption process ascribed to the synergistic effects of Ni and Co in the alloy. Additionally the electrode demonstrated good long‐term stability due to the strong interactions between exsolved nanoparticles and the parent oxide.^[28]^ Besides, interesting structural configurations seem to arise from compositions that are co‐doped with Co and Ni. It was reported that discrete Co_3_O_4_ and NiO nanoparticles could exsolve on the surface of Sr_2_CoMo_1−*x*_Ni_*x*_O_6‐δ,_ designed to be used as a supercapacitor electrode. The exsolved material showed high specific capacitance with dual energy storage mechanisms, namely a surface Faradaic reaction and an oxygen intercalation process, which were related to the presence of the exsolved nanoparticles and the increased availability of oxygen vacancies formed in perovskite, respectively.^[29]^ In catalytic applications, Ni and Co were also reported to exsolve as alloyed nanoparticles from an A‐site deficient perovskite La_0.7_Ce_0.1_Co_0.3_Ni_0.1_Ti_0.6_O_3_ and, by tracking individual nanoparticles throughout various chemical transformations, it was demonstrated that these particles have the ability to re‐shape into highly active cubes under reducing conditions. The material achieved high catalytic activity matching a noble metal commercial catalyst (Pt/Al_2_O_3_) when applied for oxidation of both CO and NO over hundreds of hours of operation.^[9c]^ More recently, a concept of endogenous‐exsolution has emerged where Ni−Co nanoparticles were exsolved both on the surface and in the bulk of the parent perovskite La_0.7_Ce_0.1_Co_0.3_Ni_0.1_Ti_0.6_O_3‐δ_. The particles seem to have Ni segregated at the core while Co was more homogenously dispersed throughout the nanoparticles. The system demonstrated high oxygen capacity, high stability over redox cycling and resistance to deactivation mechanisms like sintering and coking while still exhibiting good surface activity, all significant properties for redox cycling applications.^[8d]^

### 3.4 Other bimetallic systems

Exsolution of copper containing alloys has been reported in electrochemical applications.[^6d^, ^13d^, ^30^] For example, a composite electrode comprising of Ni_*x*_Cu_1−*x*_ alloy nanoparticles exsolved in situ on the redox‐reversible support of Nb_1.33_Ti_0.67_O_4_ was applied as a cathode for direct CO_2_ electrolysis. The ratio of the two components of the alloy appeared to have a significant effect on the electrocatalytic activity of the system with Ni_0.75_Cu_0.25_ determined as the optimal ratio.^[6d]^ Moreover, a perovskite electrode based on SrFe_0.8_Cu_0.1_Nb_0.1_O_3‐δ_ that allowed for reversible exsolution of an Fe−Cu alloy was reported to show high conductivities in both oxidising and reducing atmospheres making it a promising candidate both for SOFCs and SOECs.^[13d]^ Finally, the emergence of Cu−Co nanoparticles from a vanadate Ce_0.8_Sr_0.1_Cu_0.05_Co_0.05_VO_4‐δ_ was accompanied by a phase transition of the host into a perovskite structure Ce_0.8_Sr_0.1_Cu_0.05_Co_0.05_VO_3_. Particles seemed to be Cu‐enriched on their surface, probably due to the lower surface free energy of Cu. Such structures were found to demonstrate high activity and stability under hydrocarbon feeds in SOFC anodes;^[30a]^ the catalytic activity was thought to be provided by the Co phase while the suppression of carbon formation was attributed to the Cu‐rich surface layer.^[30a]^

Interestingly, noble metals can be paired with base metals like Ni and Fe mainly, in order to boost the catalytic activity of base metals.[^6e^, ^31^] Pt−Ni alloy (Pt_3_Ni) nanoparticles were exsolved from La_0.9_Mn_0.9_Pt_0.075_Ni_0.025_O_3‐δ_ and the newly exsolved material exhibited enhanced activity for oxygen reduction reaction (ORR) comparable to that of the commercial catalyst Pt/C, but also higher stability over Pt/C. It was also demonstrated that the enhanced activity was not solely due to the formation of Pt_3_Ni nanoparticles but also their strong interaction with the perovskite substrate.^[6e]^ Additionally, Ru−Fe alloy nanoparticles were reported to exsolve from a Sr(Ti_0.3_Fe_0.7_Ru_0.07_)O_3‐δ_ in a SOFC anode resulting in lower polarization resistance and higher maximum power density especially at low temperatures and hydrogen partial pressures, as compared to the cell using the Ru‐free perovskite anode. This was attributed to the promoting effect of the exsolved Ru−Fe nanoparticles on hydrogen adsorption which was the rate limiting step for the Ru‐free anode.^[31]^

## 4 Multielement, Nanostructured Systems

Aside from the traditional bimetallic systems, multi‐metal exsolution has been demonstrated, as well as the formation of other nanostructures, such as nano‐rods^[32]^ and nano‐fibres,^[30b]^ core–shell nanoparticles^[33]^ and even more complex heterostructures. These not only add to the structural diversity of the exsolved systems, but also to multi‐metallic systems in general, hence opening more possibilities for tailorable, enhanced catalytic performance.

**Figure 6:**
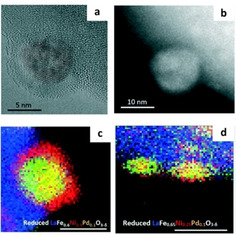
Exsolution of core–shell particles. (a) HRTEM, (b) HAADF, (c) EDS analysis of the exsolved core–shell Pd‐NiO particle. (d) Controlling the shell thickness of exsolved particles. Adapted with permission.^[33]^

Simultaneous exsolution of metals has been shown to be able to lead to particles with a core–shell structure. For instance, exsolution of Pd and Ni resulted in Pd‐NiO core–shell particles whose shell thickness and particle size could be modulated by the initial Ni doping content and reduction temperature, respectively (Figure 6). The exsolved core–shell particles were reported to be firmly socketed on the surface of the oxide support resulting in good structural stability under methane dry reforming reaction conditions.^[33]^

Interestingly, apart from “conventional” co‐exsolution, a different approach has also been used to prepare bimetallic systems. Exsolution was used in combination with infiltration of a second metal also resulting in bimetallic systems. For instance, Rh was impregnated on a three‐dimensionally ordered macroporous (3DOM) Ni‐doped perovskite support, and Ni exsolved during reduction formed alloy nanoparticles with the impregnated Rh. The catalyst showed good activity and stability for CO_2_ methanation owing to the unique 3D porous structure, the exsolved Ni−Rh alloys and their strong interaction with the support.^[34]^ Moreover, a system where Rh was infiltrated on a perovskite with *endo*‐ and *exo*‐Ni exsolved particles was used in a chemical looping methane partial oxidation process. Notably, the infiltrated Rh particles did not form any bond with the Ni particles but the modification did result in lowering the temperature at which methane was converted in dynamic temperature programmed experiments by 200 °C additionally increasing activity by 40 %.^[35]^

Reports have also demonstrated the exsolution of alloys involving three metals, and examples include Fe−Ni−Ru from LnFe_0.7−*x*_Ni_0.3_Ru_*x*_O_3‐δ_ (Ln=La, Pr)^[36]^ and Re−Ni−Fe from LaNi_0.2_Re_*x*_Fe_0.6_O_3+δ_–La_3_ReO_8_ ^[37]^ both being highly active and stable for methane dry reforming. Spinel oxides Cu_1−*x*_Ni_*x*_Fe_2_O_4_ were totally reduced to form a structure of exsolved metallic nanoparticles on a metal matrix (e.g., Cu‐rich Cu−Ni−Fe alloys on a Fe‐rich Fe−Ni−Cu matrix from Cu_0.9_Ni_0.1_Fe_2_O_4_).^[38]^ The material served as a highly active anode for electrochemical H_2_ oxidation and demonstrated much smaller anodic polarization resistance than that of a conventional NiFe alloy anode, which was ascribed to the unique alloy structure.^[38]^

**Figure 7:**
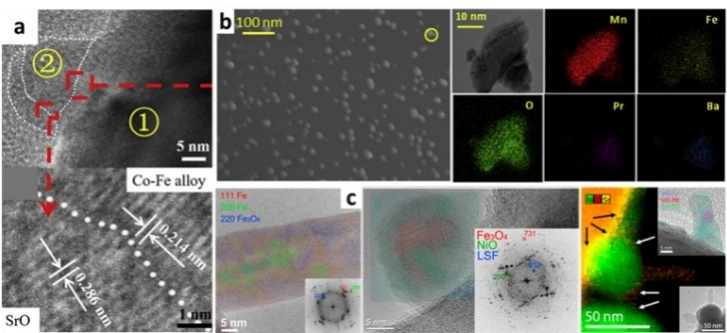
Beyond bimetallic particle exsolution. (a) Fe−Co alloy with SrO exsolved. Adapted with permission.^[39]^ (b) Fe/MnO_*x*_ nanoparticles. Adapted with permission.^[41]^ (c) Images from left to right showing the Fe nanorod exsolved from LSF in dry H_2_, the Fe−Ni alloyed particle formed on Ni–LSF in humidified H_2_, and SrO nanorod grown from Ni–LSF in dry H_2_, respectively. Adapted with permission.^[43]^

Another intriguing aspect of exsolution is that it can sometimes cause such pronounced changes to the host matrixes, that it can generate more complex structures than simple exsolved particles. For instance, exsolution of B‐site metals accompanied by the segregation of A‐site oxides at the surface of perovskites is possible, hence a SrO phase was detected to exsolve from La_0.4_Sr_0.55_Co_0.2_Fe_0.6_Nb_0.2_O_3‐δ_ matrix alongside exsolved Fe−Co nanoparticles (Figure 7 a).^[39]^ Similarly both CaO phase and Fe−Ni alloy nanoparticles were also observed on the surface of La_0.6_Ca_0.4_Fe_0.8_Ni_0.2_O_3‐δ_ after reduction.^[40]^ The two materials showed high electrolysis current density for steam splitting and CO_2_RR, respectively, attributed to the increased water/CO_2_ adsorption provided by the SrO/CaO phase and the high activity provided by the exsolved alloy nanocatalysts.[^39^, ^40^] The reduction of the A‐site deficient perovskite (Pr,Ba)_1−*x*_(Mn,Fe)O_3‐δ_ enabled exsolution of Fe/MnO_*x*_ nanoparticles (Figure 7 b) producing a very active cathode for CO_2_ electrolysis, due to the improved CO_2_ chemical adsorption and CO_2_ dissociation through electron transfer from the exsolved nanoparticles.^[41]^ Interestingly, CoO_*x*_ and La_*z*_Fe_*y*_O_*x*_ nanoparticles were exsolved within a few seconds on the surface of La_0.6_Sr_0.4_Co_0.8_Fe_0.2_O_3‐δ_ after the in situ polarization‐exsolution treatment under reductive (H_2_) atmosphere. The resulting material demonstrated enhanced performance for ORR with decreased polarization resistance, increased peak power density and good stability as compared to the pristine perovskite.^[42]^

Lastly, exsolution of two metals can also lead to other morphologies like nano‐rods and nano‐fibres. For the former, the Ni doping level in perovskites can affect the exsolution behaviour of Fe−Ni alloys, which will in turn influence the electrochemical performance of materials. Specifically, it was demonstrated that the number of exsolved Fe−Ni nanoparticles from Sr_2_Fe_1.5−*x*_Ni_*x*_Mo_0.5_O_6‐δ_ matrix increased with increasing Ni content up to *x =* 0.2 while the particles remained round and of the order of about 25 nm as compared to the nanorod‐like Fe−Ni alloys that formed when *x* > 0.2. The self‐assembly of nanoparticles into nanorods seem to have been facilitated by the excessive amount of Fe and Ni ions that left the host. Thus, *x =* 0.2 was found to be the optimal Ni doping content for the highest activity in steam electrolysis.^[32]^ Additionally, a study focused on exsolution from LSF under different conditions observed that when treated in dry H_2_ Fe exsolved in the form of nanorods (Figure 7 c, left), which indicates that the exsolution process was limited by oxygen removal rather than the transport of Fe cations. If the transport of iron species is much faster than oxygen removal itself only one nucleus is formed which acts as a “iron drain” leaving the Fe‐containing structure no other way than to grow but in a rod like manner. No exsolution was observed under humidified H_2_ but impregnating Ni on LSF considerably changed the exsolution behaviour of the cations and in this case Fe was found to exsolve from Ni–LSF in humidified H_2_ but formed Fe−Ni alloys instead of nanorods (Figure 7 c, middle). When treated in dry H_2_, in addition to the Fe−Ni particles, exsolution of SrO nanorods (Figure 7 c, right) was also observed which seemed to be triggered by the presence of Ni due to the dissociative hydrogen activation around Ni particles.^[43]^ Moreover, another study reported the preparation of a high‐performance composite anode involving exsolved Fe−Cu in the form of nano‐fibres. The solid state method used allowed for a nominal oxide La_0.5_Sr_0.6_Fe_0.8_Cu_0.15_Nb_0.05_O_3‐δ_ to self‐assemble into a mixture of a single perovskite (Sr_0.4_La_0.6_FeO_3_‐type) and a RP‐type layered perovskite (SrLaFeO_4_‐type) and hence create a hetero‐interface which, in addition to the unique nano‐fibre morphology of the exsolved Fe−Cu, mitigated the coarsening of substrate grains and sintering of the exsolved nanoparticles.^[30b]^

## 5 Summary and Outlook

Exsolution is a relatively new research hotspot which can be traced back to 2002.^[12a]^ Exsolution of bimetallic species has gained increased attention even more recently. Herein we have reviewed the, approximately, 70 studies published so far, on bimetallic exsolution and identified research trends in this area. It has been demonstrated that exsolution can endow bimetallic particles with advantages in the terms of high catalytic activity, improved electrochemical properties, prolonged durability and strong resistance to deactivation in a wide range of applications mainly including electrochemistry and catalysis. Moreover, alloying such particles also allows for tuning of the adsorption properties, catalytic activity and stability of the exsolved particles due to the synergistic effects of different metals. However, most of these studies reported so far are application‐oriented while less attention has been paid on the principles of material design. In order to further improve the performance of the exsolved bimetallic systems and unleash their full potential, some challenging issues should be resolved, including but not limited to the ones identified below.

Firstly, reduction conditions, initial composition and defects of the parent materials can directly affect the ratio of the metals of the exsolved alloys which in turn could determine catalytic activity and selectivity. Taking this into consideration more systematic study is still required of this aspect to allow for fine tuning of the chemical nature of the exsolved alloys and indeed to improve understanding of the exsolution process itself.

Secondly, as compared to the exsolved monometallic particles that are usually in the form of simple spheres or ellipsoids, exsolution of different metals offers richer diversity of structures which can bring additional emergent functionalities to materials. However, studies on this aspect are still very limited, and creating, characterising and applying novel structures of exsolved bimetallic materials is highly desirable.

Finally, the importance of modelling studies should be highlighted, which combined with the experimental observations will strengthen the understanding of the mechanism of bimetallic exsolution ultimately allowing for full control over the design of such materials.

## Conflict of interest

The authors declare no conflict of interest.

## Biographical Information

*Chenyang Tang received his bachelor's degrees in chemical engineering from a joint program between University of Birmingham (UK) and Beijing University of Chemical Technology (China) in 2015. In September of the same year, he joined The Application of Ion Transport Group at Newcastle University where he received his PhD in 2020 and then continued to work as a postdoctoral research associate under the supervision of Prof. Ian Metcalfe. His research interests are in the development and application of new nanostructured materials for different catalytic processes and he is currently focusing on emergent nanomaterials*.

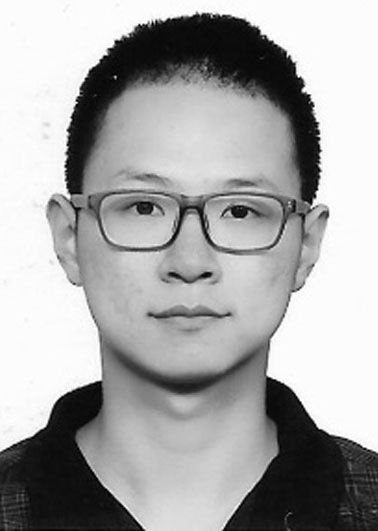

## Biographical Information

*Kalliopi Kousi received her degree in Chemistry from the University of Patras, Greece in 2011. She has an MRes (2013) and a PhD (2016) on ‘Catalysis for Environmental Protection and Clean Energy Production’ and has worked as post‐doctoral research associate at the department of Chemical Engineering focusing on catalysts for LPG conversion. She joined The Application of Ion Transport Group at Newcastle University as a post‐doctoral research associate in April 2017. Her research interest lies in new materials and structures for different catalytic applications, currently focusing on emergent nanomaterials for energy conversion processes*.

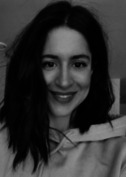

## Biographical Information

*Dragos Neagu obtained his degree in chemical engineering from University Politehnica of Bucharest, Romania, in 2008. He received his PhD on materials and devices for electrochemical energy conversion from the University of St Andrews, UK in 2013. He then worked as a postdoctoral researcher at the University of St Andrews and Newcastle University. In 2020 he joined the University of Strathclyde as Lecturer in the Department of Chemical and Process Engineering. His research interests are in the design, preparation, multiscale characterization and application of nanostructured, functional materials for renewable energy conversion technologies*.

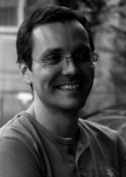

## Biographical Information

*Ian Metcalfe obtained his first degree in chemical engineering from Imperial College and then studied at Princeton University. He is currently Professor of Chemical Engineering at Newcastle University. Ian is a Fellow of the Institution of Chemical Engineers and a Fellow of the Royal Society of Chemistry. He was elected a Fellow of the Royal Academy of Engineering (RAEng) in 2012. He currently holds an RAEng Chair in Emerging Technologies. His research concerns the thermodynamics of chemical conversion with an emphasis on energy processes. He has a particular interest in membrane processes, solid–gas reactions and solid state chemistry*.